# Papillary fibroelastoma, unusual cause of stroke in a young man: a case report

**DOI:** 10.1186/s13019-017-0592-6

**Published:** 2017-05-19

**Authors:** Elisabetta Grolla, Michele Dalla Vestra, Giampaolo Zoffoli, Riccardo D’Ascoli, Adriana Critelli, Rocco Quatrale, Domenico Mangino, Fausto Rigo

**Affiliations:** 1 Department of Cardiology, Ospedale dell’Angelo, Venezia-Mestre, Italy; 2 Department of Internal Medicine, Angiology Unit, Ospedale dell’Angelo, Venezia-Mestre, Italy; 3 Department of Cardiac Surgery, Ospedale dell’Angelo, Venezia-Mestre, Italy; 4 Department of Neurology, Ospedale dell’Angelo, Venezia-Mestre, Italy

**Keywords:** Papillary fibroelastoma, Cardiac tumors, Cardioembolic stroke, Cerebrovascular disease, Case report

## Abstract

**Background:**

Papillary fibroelastoma is the third most common primary benign tumor with an incidence of up to 0.33% in autopsy series; it accounts for approximately 75% of all cardiac valvular tumors.

**Case presentation:**

We describe a rare case of a 28-Year-old man that while playing football, had a sudden onset of neurological deficit: aphasia, right hemiparesis and right facial numbness. Transthoracic echocardiography (TTE) showed a 10x10 mm mass attached to the anterior mitral valve leaflet. The patient was treated surgically for the prevention of further embolic complications. Histologic examination of the resected mass revealed a papillary fibroelastoma. It is the third most frequent primary cardiac tumor, after myxoma and fibroma, and the most common primary tumor of heart valves. Despite the benign nature of this tumor, it carries very high risk of embolic complications. The successful complete resection of the papillary fibroelastoma is curative and the long-term postoperative prognosis is excellent.

**Conclusions:**

Differential diagnosis of cardiac masses requires clinical informations, laboratory tests, blood cultures and appropriate use of imaging modalities. Papillary fibroelastoma is a potential cause of embolic stroke in the young. The prompt surgical excision of papillary fibroelastoma is curative and the long-term postoperative prognosis is excellent.

## Background

Papillary fibroelastoma is the third most common primary cardiac benign tumor with an incidence of up to 0.33% in autopsy series; it accounts for approximately 75% of all cardiac valvular tumors and affects men and women equally with a mean age of 60 years at diagnosis. Despite the benign nature of this tumor, it carries very high risk of embolic complications. Here we described a case of stroke in a 28-year-old man due to cerebral embolization originated from a cardiac papillary fibroelastoma.

## Case presentation

A 28-years-old man was admitted to the Emergency Department of our Hospital for the sudden onset of aphasia, right facial numbness and right hemiparesis, while playing football. He was not an athlete and never performed a medical examination for sports fitness.

**Fig. 1:**
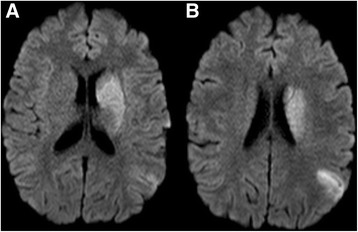
Brain Magnetic Resonance shows areas of recent ischemia in the left cerebral hemisphere with more extensive involvement of the putamen, globus pallidus and temporal and parietal cortical-subcortical posterior lobes

**Fig. 2:**
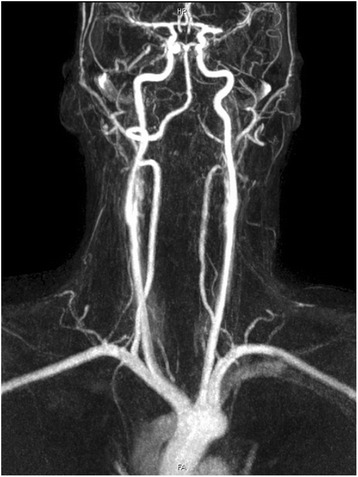
Epiaortic Magnetic Resonance Angiography shows an occlusion at the origin of the *left* middle cerebral artery

Nothing of significant emerged in the past medical history. Physical examination confirmed the neurological deficit, blood pressure was 120/80, heart rate 90 bpm, no carotid bruit detected. Blood tests (blood count, kidney and liver function, coagulation and electrolytes) were normal. Electrocardiography (ECG) showed normal sinus rhythm. Computer Tomography (CT) of the brain, performed in urgency, was normal. A Magnetic Resonance (MRI) of the brain showed areas of recent ischemia in the left cerebral hemisphere with more extensive involvement of the putamen, globus pallidus and temporal and parietal cortical-subcortical posterior lobes (Fig. 1 panel a and b). Smaller areas of similar meaning were detectable always on the left, adjacent parietal-occipital sulcus and on the right in the frontal and cerebellar regions. Some small areas compatible with vascular outcomes were found in the cerebellum. Magnetic Resonance Angiography (MRA) showed thrombosis of the left middle cerebral artery (Fig. 2). The patient was hospitalized in Stroke Unit and in accordance with the guidelines was treated with thrombolysis with r-TPA (0.9 mg/kg). Mechanical thrombectomy was not considered as the first choice of treatment since the patient did not have contraindications to thrombolysis; given the good clinical response to thrombolytic treatment, mechanical thrombectomy was not considered as an adjuntive treatment either.

**Fig. 3:**
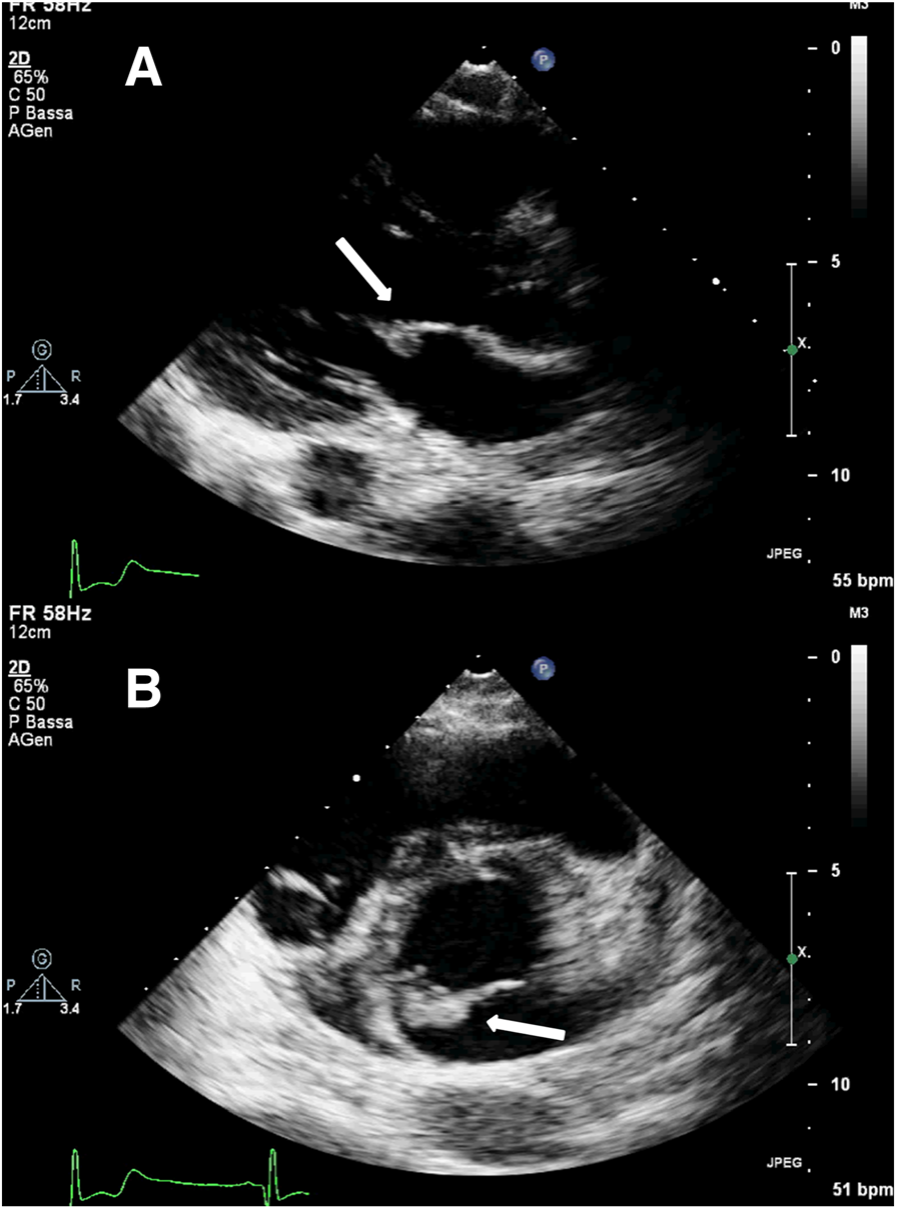
Transthoracic echocardiogram. The long axis (panel **a**) and short axis views (panel **b**) show the hyperechogenous mass (*white arrow*) attached to atrial side of A3 scallop of the anterior mitral leaflet

**Fig. 4:**
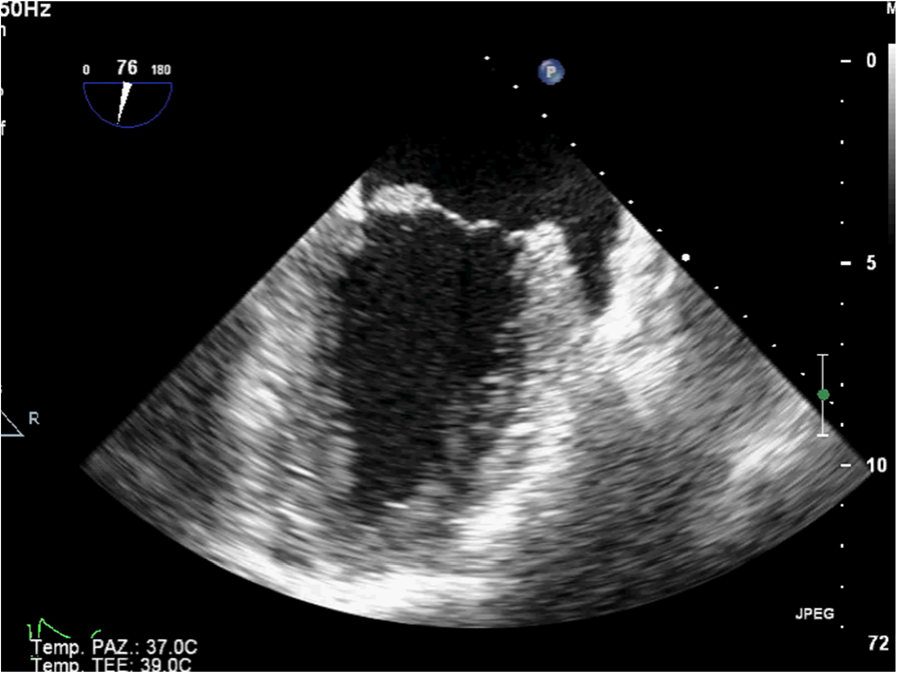
Transesophageal echocardiogram. The intercommissural view (76°) shows a mobile nodular mass, measuring 1 × 1 cm, on the anterior leaflet of the mitral valve

**Fig. 5:**
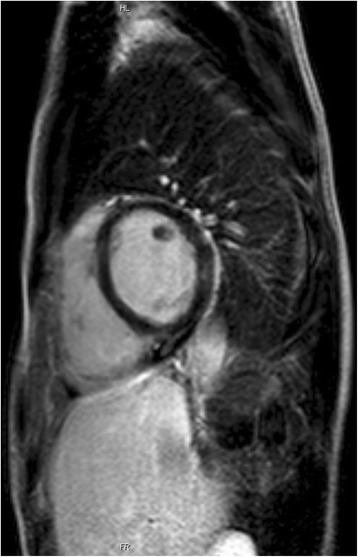
The Cardiac MRI shows a round mass adhering to the mitral valve

Transthoracic echocardiography (TTE) showed a 10 × 10 mm mass attached to the anterior mitral valve leaflet, fluttering into the cardiac chambers. (Fig. 3 panel a and b). There were no evidence of valvular stenosis, regurgitation, or left ventricular outflow tract obstruction and left ventricular function was normal. A transesophageal echocardiography (TEE) was also performed: it confirmed the presence of a hyper echogenic nodular and mobile mass, attached to the atrial side of A3 scallop of the anterior mitral leaflet; it also allowed to rule out other causes of embolism such as patent foramen oval or cardiac thrombosis (Fig. 4). A cardiac MRI confirmed the presence of the lesion, with a maximum size equal to about 1 cm (Fig. 5). ECG monitoring during hospitalization in Stroke Unit and the Holter ECG revealed no arrhythmias.

**Fig. 6:**
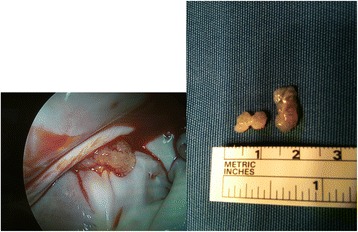
In the intraoperative view the lesion appears as a white round mass, without a peduncle. The gross specimen of the mass demonstrated gelatinous appearance and smooth surface, with many white papillary fragments with frond-like projections, typical of papillary fibroelastoma

The decision was to perform the excision of the mass. We considered the possibility to remove it in the cardiac catheterization laboratory but the mass was apparently too big and indented to be removed with out risk of embolization. To avoid it we decided to perform an open heart surgical excision. The operation was performed under normothermic cardiopulmonary bypass using ascending aortic and bicaval cannulation. After cardiac arrest with antegrade cardioplegia, left atrium was opened A 1-cm, gelatinous and solid looking mass was found attached to the anterior leaflet of mitral valve near the posteromedial mitral commisure. It was resected with its stalk (Fig. 6). Post-operative TEE confirmed normal valvular functions and absence of residual left atrial mass. The histological examination of the tissue revealed a papillary fibroelastoma. The early postoperative period was uncomplicated, and the patient was discharged on postoperative day-10. Actually remains a permanent nuanced right hemisyndrome. A 1-month postoperative echocardiography control showed perfect valve function with no residual mitral regurgitation or stenosis.

## Discussion

Here we described a rare and very special case of stroke in a 28-year-old man due to cerebral embolization originated from a cardiac papillary fibroelastoma. Stroke in the young, with no obvious risk factors, usually requires extensive evaluation: it is important to look for secondary cause of cerebral ischemia, searching carefully especially diseases with potential cardioembolic.

Papillary fibroelastoma is the third most common primary benign tumor with an incidence of up to 0.33% in autopsy series; it accounts for approximately 75% of all cardiac valvular tumors and affects men and women equally with a mean age of 60 years at diagnosis. According to epidemiological data described cases rarely involve young patients [1, 2]. Althought papillary fibroelastomas are histologically benign neoplasms, they may result in life-threatening complications if valve obstruction or systemic embolization occurs, as described in our patient.

Most patients are asymptomatic, but some patients may experience cerebral embolic symptoms, such as stroke or transient ischemic attack or angina, acute coronary syndrome, myocardial infarction or death from coronary ostial obstruction [3–25]: transient ischemic attack/cerebrovascular accident is considered by far the most common presentation of papillary fibroelastoma [26]. This tumor has a predilection for the left side of the heart: the aortic valve is the predominant site involved, followed by mitral leaflets. Grossly, papillary fibroelastoma resemble a “sea anemone”. This tumor usually has a gelatinous membrane on the surface and a stalk with multiple delicate papillary projections, best appreciated by immersing the specimen in water [27]. Microscopically, it is characterized by a collection of avascular fronds of dense connettive tissue lined by endothelium and may arise from any endocardial surface. Embolic fragments may originate from the tumor itself and this occurs because of its very friable and soft texture, or from surface formation of platelet and fibrin thrombi [28]. Most are solitary and small, some are mobile and appear more likely to give rise to embolism. Tumor mobility has been described to be an independent predictor of death or non-fatal embolization [29]. Echocardiography is the preferred means for evaluation of papillary fibroelastomas [30]. Due to their small size generally 0.5 to 2.0 cm in diameter and their valvular involvement, papillary fibroelastoma may be difficult to distinguish from valvular vegetation. For this reason, clinical informations, laboratory tests and blood cultures are extremely important for differential diagnosis. The differential diagnosis includes the presence of mixoma, lipoma, rhabdomyoma or amorphous tumors. Since symptomatic papillary fibroelastoma carries a definite risk of severe complications, aggressive surgical management is recommended, irrespective of the tumor’s size or the patient’s symptoms [26], the successful complete resection of the papillary fibroelastoma is curative and the long-term postoperative prognosis is excellent. The patients who are not surgical candidates could be offered long-term oral anticoagulation, although non randomized controlled data are available on its efficacy.

## Conclusions

Echocardiography transthoracic and transesophageal are essential investigations in young patients with stroke. Differential diagnosis of cardiac masses requires clinical informations, laboratory tests, blood cultures and appropriate use of imaging modalities. Papillary fibroelastoma is a potential cause of embolic stroke in the young. The prompt surgical excision of papillary fibroelastoma is curative and the long-term postoperative prognosis is excellent.
